# Pathogenicity and Virulence of Ebolaviruses with Species- and Variant-specificity

**DOI:** 10.1080/21505594.2021.1898169

**Published:** 2021-03-18

**Authors:** Satoko Yamaoka, Hideki Ebihara

**Affiliations:** Department of Molecular Medicine, Mayo Clinic, Rochester, USA

**Keywords:** Ebola virus, Sudan virus, Bundibugyo virus, Taï Forest virus, Reston virus, virulence, case fatality rates

## Abstract

Ebola virus (EBOV), belonging to the species *Zaire ebolavirus* in the genus *Ebolavirus*, causes a severe febrile illness in humans with case fatality rates (CFRs) up to 90%. While there have been six virus species classified, which each have a single type virus in the genus *Ebolavirus*, CFRs of ebolavirus infections vary among viruses belonging to each distinct species. In this review, we aim to define the ebolavirus species-specific virulence on the basis of currently available laboratory and experimental findings. In addition, this review will also cover the variant-specific virulence of EBOV by referring to the unique biological and pathogenic characteristics of EBOV variant Makona, a new EBOV variant isolated from the 2013–2016 EBOV disease outbreak in West Africa. A better definition of species-specific and variant-specific virulence of ebolaviruses will facilitate our comprehensive knowledge on genus *Ebolavirus* biology, leading to the development of therapeutics against well-focused pathogenic mechanisms of each Ebola disease.

## Introduction

Ebola virus disease (EVD) is an acute viral zoonotic disease with high case fatality rates (CFRs) reaching as high as 90%. The disease is characterized by sudden onset of high fever, gastrointestinal symptoms including diarrhea and vomiting, respiratory symptoms, rash, conjunctival injection, and hemorrhagic manifestations. Fatal cases terminate in hypovolemic shock and multiorgan failure [1,2]. Ebola virus (EBOV), the causative agent of EVD, is a negative-sense, single-stranded RNA virus in the genus *Ebolavirus* of the family *Filoviridae*. In the genus *Ebolavirus*, there have been six virus species classified that each have a single type virus [1,3]. Among them, EBOV is often referred to as the most virulent ebolavirus, in large part because EBOV has been responsible for the majority of Ebola disease outbreaks thus far, including several epidemics with significantly high CFRs (>70%) [1,4,5]. Aside from the epidemiological aspect, some laboratory and experimental findings also indicate that virulence/pathogenicity of ebolaviruses in humans differs among viruses belonging to each distinct species. In this review, we aim to define the virus species-specific difference on the basis of laboratory and experimental findings, including molecular insights. We first outline current knowledge on EBOV pathogenesis based on three aspects, such as clinical, *in* *vivo*, and *in vitro* studies. We then interpret the distinct virulence profiles among the ebolaviruses belonging to the different species and further discuss the research gaps in our understanding of this species-specific pathogenicity of ebolavirus in humans. Finally, this review deliberates recent findings of virulence of the EBOV-Makona variant, a newly identified EBOV variant from the largest EVD outbreak in West Africa in 2013–2016, which exhibits unique virulence characteristics compared to the known EBOV variants/strains.

## Molecular biology of ebolaviruses

**Figure 1.**
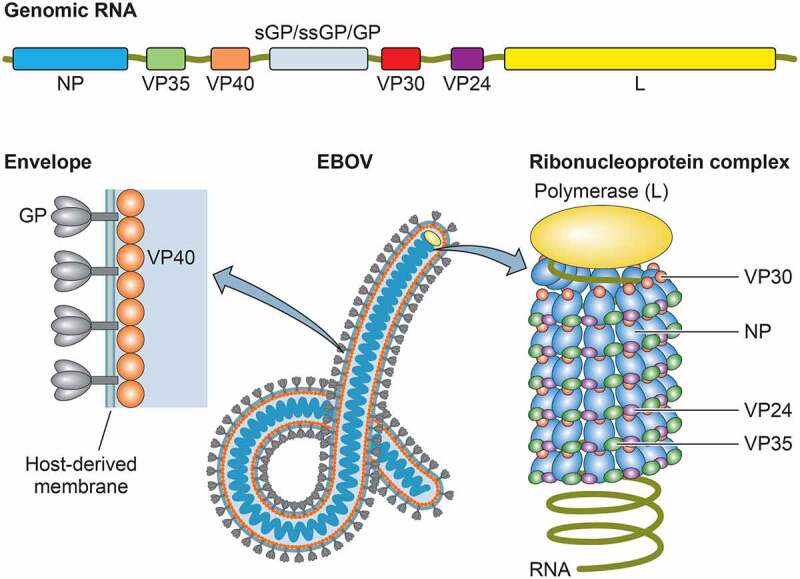
Genomic organization and viral particle of ebolaviruses. The single-stranded, negative-sense genome consists of a linear RNA molecule of approximately 19 kb that is composed of seven genes: NP, VP35, VP40, GP, VP30, VP24, and L. The viral particles are filamentous in shape, consisting of a nucleocapsid core surrounded by a viral matrix protein VP40 and a host-derived envelope studded with GP spikes

The EBOV comprises negative-sense, single-stranded RNA genome that consists of seven genes, encoding the nucleoprotein (NP), virion protein 35 (VP35), VP40, the glycoprotein (GP), VP30, VP24, and the RNA-dependent RNA polymerase (L) [6]. Additionally, soluble GP and small soluble GP are encoded by the GP gene [7–9]. The termini of the genome comprise a 3′ leader and a 5′ trailer that contain replication/transcription promoters and genome packaging signals. The EBOV particles possess a ribonucleoprotein (RNP) complex, consisting of the viral genome RNA encapsidated in the NP, VP35, VP30, VP24, and L, surrounded by a viral matrix protein VP40 and a host-derived envelope studded with GP spikes (Figure 1). The L, along with VP35, a polymerase cofactor, and viral transcription factor VP30 drive replication of the viral genome and transcription of the genes [10,11]. While VP40 is essential for virion assembly and budding [12–14], GP mediates viral entry, including attachment to receptor molecules and membrane fusion [15,16]. The VP24 plays a key role in condensing viral nucleocapsids, which is important for efficient packaging of genome/nucleocapsid into the virion [17–22].

## Taxonomy of ebolaviruses

Ebolaviruses belong to the family *Filoviridae,* which comprises six virus genera, including *Ebolavirus, Marburgvirus, Cuevavirus, Dianlovirus, Striavirus*, and *Thamnovirus* [3]. In the genus *Ebolavirus*, six species that each have a single type virus have been identified to date: *Zaire ebolavirus* with type virus Ebola virus (EBOV), *Sudan ebolavirus* with type virus Sudan virus (SUDV), *Bundibugyo ebolavirus* with type virus Bundibugyo virus (BDBV), *Tai Forest ebolavirus* with type virus Taï Forest virus (TAFV), *Reston ebolavirus* with type virus Reston virus (RESTV), and *Bombali ebolavirus* with type virus Bombali virus (BOMV) [1,3]. The average amino acid identity among all viral proteins between viruses belonging to genus *Ebolavirus* ranges from 60% to 80% [23].

Note that the term “species” used throughout this review refers to “virus species.” In addition, we use the term “Ebola disease” to describe all diseases caused by the infection of viruses belonging to genus *Ebolavirus*, and the terms “Ebola virus disease (EVD),” “Sudan virus disease (SVD),” and “Bundibugyo virus disease (BVD)” to describe each Ebola disease that is subcategorized according to its causative agent [24].

## Outbreaks and CFRs for ebolavirus infections

**Figure 2.**
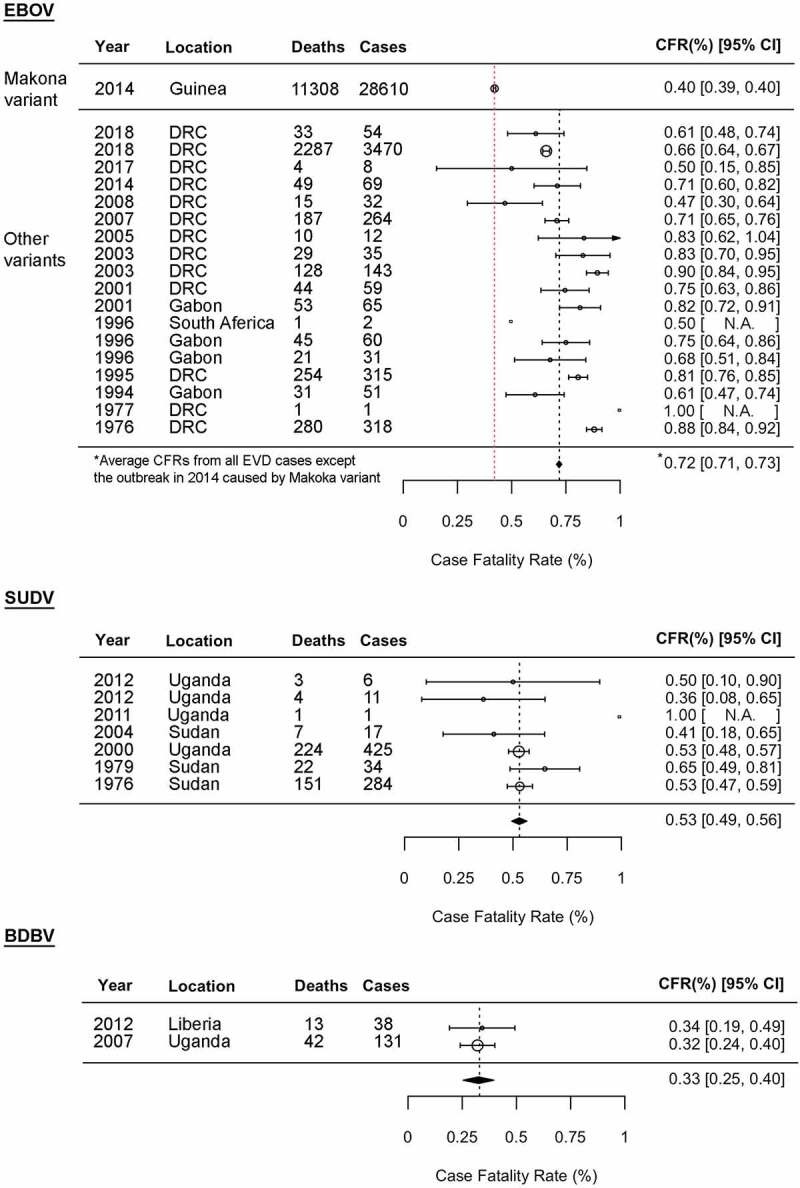
Ebola disease outbreaks and case fatality rates (CFRs) of EBOV, SUDV, and BDBV infections. Forest plot shows the average CFRs and 95% confidence intervals (CIs) for the Ebola disease outbreaks by fixed-effect model with inverse-variance weighting. Meta-analysis was performed using R software with the metaphor package. The CFRs from the 2013–2016 EVD outbreak caused by EBOV-Makona variant is separately calculated from the average CFRs from all other EVD cases. N.A: not available due to small sample size

Although all ebolaviruses, except for BOMV, are known to infect humans, CFRs of ebolavirus infections vary, ranging from no fatalities (0% CFR) in RESTV and TAFV infections to reaching up to 90% of EBOV infection (Figure 2) [5]. EBOV has been responsible for the majority of Ebola disease outbreaks to date since its first discovery in 1976, including the largest EVD outbreak in history that occurred in West Africa in 2013–2016 [1,4,5]. Despite its magnitude, the CFR for the 2013–2016 West Africa EVD outbreak was 40%, which was exceptionally low compared to other EVD outbreaks reported so far. Multiple factors, including a better international outbreak response, such as robust clinical trials and clinical interventions, likely contributed to the observed low CFRs in the West Africa EVD outbreak. A possible involvement of a virological factor in the low CFRs will be discussed in Section “Pathogenesis of EBOV-Makona variant”. The average CFR for EVD excluding the 2013–2016 EVD outbreak is 72% (Figure 2).

SUDV was first discovered in 1976, and thus far has been responsible for seven outbreaks [25,26]. The largest SVD outbreak in 2000 resulted in more than 400 human infections with a CFR of 53%. Compared to EBOV and SUDV, BDBV is often referred to as a less virulent ebolavirus. Two BVD outbreaks have been reported in 2007 [27] and 2012 [28–30], with culminating average CFR as 33%. To date, there has been only one confirmed case of severe, but nonfatal, TAFV infection in humans reported in 1994 [31,32]. A total of 74 persons were defined as direct contact persons with the patient, but all were shown as seronegative for ebolaviruses [32].

While EBOV, SUDV, BDBV, and TAFV cause often severe/fatal diseases in humans, RESTV is apparently apathogenic to humans. There has been no evidence showing that RESTV is associated with human disease since its first discovery in 1989 [33], despite the apparent occurrence of human infections evidenced by seropositive titers of RESTV-specific antibody that relates to several RESTV epizootics in nonhuman primates (NHPs) or domestic pigs [34–37].

BOMV was first discovered in bats in 2018 [38], followed by the second identification in bats in 2019 [39]. The potential of this virus to infect/cause disease in humans is currently unknown.

## Pathogenesis of EVD and its molecular mechanisms

Studies on ebolavirus pathogenesis have mostly been focused on EBOV and its disease. Owing to numerous studies performed during the 2013–2016 West Africa EVD outbreak, detailed clinical insights on EVD have also become available. In this section, we will provide current knowledge on EVD pathogenesis at both *in vivo* and molecular levels. Note that we will mainly focus on the aspects of which insights are also available in studies of other ebolavirus species. The comparison between EVD (EBOV) and other Ebola diseases (other ebolaviruses) will be provided in Section “Comparison of pathogenesis among viruses belonging to genus *Ebolavirus*”.

### Clinical and laboratory findings on EVD

The initial symptoms of EVD are a nonspecific febrile illness followed by gastrointestinal manifestations [1,2,40–50]. Some respiratory symptoms, hiccup, conjunctival injection, and macropapular rash are also often reported. Hemorrhagic signs are observed in approximately half of the infected persons, with no clear correlation between bleeding and disease severity [44,51,52]. The mean incubation period for EVD from contact exposure to the onset of symptoms is 7.34 ± 1.35 d [53]. In fatal cases, death occurs 6–16 d after the onset of symptoms as a result of hypovolemic shock and multiorgan failure [2].

Acute, robust, and systemic viral replication is the most consistent observation in severe/fatal EVD. Viral antigen and nucleic acid can be detected in patients’ blood from d 1 to 3, peaking at around 6–7 d post symptom onset, and remain high throughout the course of the disease in fatal cases [1,42,54,55]. A number of clinical studies have shown a strong correlation between viremia titer and EVD fatality [40–42,44,50,55–57]; high blood viral load (≥10^6^ copies/ml) was found to be predictive of the fatal outcome [41,44]. Markedly elevated levels of alanine aminotransferase (ALT), which is a biochemical marker for liver integrity, and aspartate aminotransferase (AST), which is a biochemical marker for liver, heart skeletal muscle, kidney, brain, and red blood cell integrity, are major serum biochemical signatures seen in severe/fatal EVD, indicating serious tissue damage due to EBOV infection [44].

Over-activated, detrimental pro-inflammatory cytokine/chemokine responses, such as the so-called cytokine/chemokine storm, are also one of the most common patho-immunological features in severe/fatal EVD [42,55,57–65]. Massive production of multiple pro-inflammatory cytokines [e.g. interleukin 1 beta (IL-1β), IL-6, tumor necrosis factor alpha (TNF-α)] and pro-inflammatory chemokines [e.g. IL-8, macrophage inflammatory protein 1 alpha (MIP-1α), MIP-1β, monocyte chemotactic protein 1 (MCP-1), macrophage colony-stimulating factor (M-CSF), interferon gamma-induced protein 10 (IP-10)] are detected in deceased EVD patients. This over-activation of pro-inflammatory mediators results in systemic inflammatory response syndrome (SIRS) that also triggers mixed/compensatory anti-inflammatory response syndrome (MARS/CARS). Indeed, up-regulation of anti-inflammatory cytokines (e.g. IL-1RA, IL-10, sTNF-RI, sTNF-RII) are also observed in EVD patients. This aberrant immunological status, such as SIRS coupled with MARS/CARS, is characteristic of classical bacteria sepsis [66] and has been known to play a significant role in induction of endothelial dysfunction, vascular leakage, and coagulation abnormalities.

Uncontrolled pro- and anti-inflammatory cytokine responses are also linked to the impairment of host adaptive immunity seen in fatal EVD patients. Ruibal et al. have demonstrated that, compared to survivors, EVD fatalities had a higher percentage of T cells expressing inhibitory molecules CTLA-4 and PD-1, despite no difference in the activation status of T cells between fatalities and survivors [65]. Moreover, while strong EBOV-specific T cell responses were detected in EVD survivors [67–69], antigen-specific T cell responses were found to be very rare in fatal cases [65], indicating nonspecific, dysfunctional T cell activation in EVD fatalities. Accordingly, the humoral response mediated by B cells was also impaired in fatal EVD cases [55,61,70]. In sum, robust viral replication and immune dysfunction are key hallmarks in severe/fatal EVD patients, significantly contributing to EVD pathogenesis.

### EBOV pathogenesis studied in animal models

Animal models of EBOV infection have so far been developed in NHPs and small animal models, such as mice, guinea pigs, Syrian golden hamsters, and ferrets. Among them, NHPs are considered the “gold standard” animal model since they are highly susceptible to the infection of wild-type EBOV (WT-EBOV) that have been isolated from human samples. Nearly all clinical and pathological features of severe/fatal EVD in humans can be recapitulated in NHPs, including high viremia, strong cytokine/chemokine response, coagulopathy, rash, and hemorrhagic signs [71–75]. Cynomolgus and rhesus macaques have been the most commonly used NHPs for EBOV infection; disease progression in cynomolgus macaques after EBOV infection is slightly faster than that in rhesus macaques [6]. Pathological studies in EBOV-infected NHPs have clearly demonstrated that cells of the mononuclear phagocytic system (MPS) (i.e. monocytes, macrophages) and dendritic cells (DCs) are the initial target cells [71,73,76] that migrate to target organs (e.g. liver, lymph nodes, spleen), resulting in the efficient transmission and replication of the virus [77]. In addition to NHPs, lethal infection with WT-EBOV can also be achieved in ferrets with hallmark pathological features of EVD, including fever, petechial rashes, hemorrhage, and coagulopathy [78].

Unlike NHPs and ferrets, immunocompetent rodents show no or very mild signs of disease after WT-EBOV infection. Thus, immunocompetent rodent models rely on using rodent-adapted virus strains that have been established as a consequence of serial passage of the virus in the host animals, such as mice or guinea pigs, leading the virus to acquire the ability to cause uniformly lethal infection in rodents [79,80]. Two of the mouse-adapted EBOV strains (MA-EBOV) have been developed based on EBOV variant Mayinga [81] and Makona [82], and four of the guinea pig-adapted EBOV strains (GPA-EBOV) are available [79,80,83–86]. Rodent-adapted EBOV targets the same cells/tissues as the WT-EBOV infection in the lethally susceptible animals (i.e. NHPs, humans) and develops high viremia in the infected host rodents [81,83,87]. MA-EBOV can also cause lethal infection in Syrian golden hamsters with several critical EVD signatures including cytokine/chemokine responses and coagulopathy [88]. A collaborative cross (CC) resource recombinant inbred (RI) intercrossed (CC-RIX) mouse model with MA-EBOV infection has also been developed, which shows severe coagulopathy that is not evident in other conventional laboratory mouse strains [89].

Furthermore, several lethal immunodeficient rodent models with WT-EBOV infection have been developed and widely used for EBOV pathogenesis studies. In addition to the classical immunodeficient mice, such as type I interferon (IFN)-deficient mouse strains (i.e. IFN-α/β receptor knockout (IFNAR^−/-^) mice [90,91], STAT1 knockout (STAT1^−/-^) mice [92]), and adaptive immunity-deficient mouse strain (i.e. SCID mice) [90], several human immune system (HIS) mice, generated by xeno-engrafting human immune cells or tissues and/or their progenitors into immunodeficient mice, have also been utilized for studies of EBOV pathogenesis [93–100]. The use of HIS mice allows for the examination of the cell/organ-specific human immune response and its interaction with virus *in vivo*, thus they are considered a valuable tool for studying EVD pathogenesis.

### Molecular pathogenic mechanisms of EBOV infection

Among all EBOV proteins, VP35, VP24, and GP have been considered as the main virulence factors of EBOV. VP35 functions as an IFN antagonist, which inhibits host type I IFN-α/β induction, and also inhibits the phosphorylation of double-stranded RNA-activated protein kinase R that mediates cellular antiviral responses [101–110]. The introduction of mutations disabling the VP35’s IFN-antagonistic function results in attenuation of virulence in animal models including NHPs [103,108,111–113]. While VP35 counteracts the type I IFN induction pathway, VP24, the other EBOV IFN antagonist, blocks the type I IFN signaling cascade by blocking karyopherin α (KPNA)-mediated nuclear translocalization of phosphorylated STAT1 homodimers or STAT1-STAT2 heterodimers [114–117]. The restriction of STAT nuclear translocalization by VP24 results in reduced transcriptional activation of IFN-stimulated genes (ISGs), preventing the establishment of an antiviral state in the host cells. The significant role of VP24 for the acquisition of virulence in rodents has been reported [79,84–86,118–120]. VP35 and VP24 also contribute to suppression of DC activation/maturation, resulting in induction of impaired cell-mediated responses [121–124], as also seen in EBOV-infected DCs [125,126].

The GP has been shown to interact with host toll-like receptor 4 (TLR4) leading to the activation of pro-inflammatory responses via the NF-κB pathway [127–131]. In addition, the shed GP, which is a truncated version of the surface GP cleaved by cellular metalloprotease TACE, has also been shown to induce immune activation in a TLR4-dependent manner, aside from its antibody-neutralizing activity and endothelial-permeabilizing activity [132–134]. Moreover, a recent study demonstrated the interaction of virion-associated phosphatidylserine with Tim-1 and showed its significant role in T-cell activation, production of pro-inflammatory mediators, and pathogenesis in the animal model [135].

## Comparison of pathogenesis among viruses belonging to genus *Ebolavirus*

**Figure 3.**
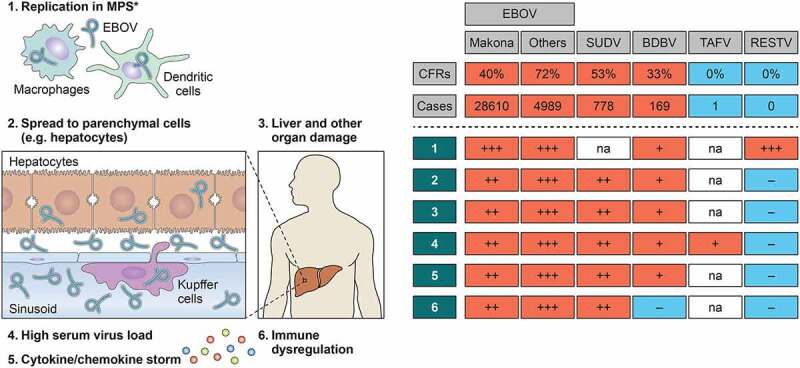
Species-specific and variant-specific virulence of ebolaviruses. Viral ability to spread from macrophages to parenchymal cells, leading to severe organ damage, is one of the key phenotypic features determining ebolavirus pathogenicity. The numbers 1–6 shown in a table on the right are correlated with those shown in a figure on the left. *Including Kupffer cells. na: not available

The CFRs of Ebola diseases in humans can range from no fatalities (0% CFR) in RESTV and TAFV infections to reaching up to 90% of EBOV infection (Figure 2). Although clinical insights on the SUDV, BDBV, TAFV, and RESTV infections in humans are limited, there have been intriguing experimental *in vivo and in vitro* studies available that examine differing pathogenesis of these viruses. Several small animal models (i.e. ferrets, humanized mice) that propagate lethal SUDV, BDBV, TAFV, and RESTV infections have also been developed, greatly facilitating the studies on ebolavirus pathogenesis. In this section, we will compare the pathogenesis of EVD (discussed in Section “Pathogenesis of EVD and its molecular mechanisms”) with other Ebola diseases and define species-specific differences on the basis of experimental findings (Table 2 and Figure 3).

### Disease courses and fatalities in animal models

Differing disease severity and lethality associated with each ebolavirus infection have been demonstrated by several animal studies (Table 1). In the case of NHP models, EBOV causes 100% lethality in cynomolgus macaques by 5–7 d [136–139] and in rhesus macaques by 6–9 d [74,136,140] after infection with a challenge dose of 10^3^ PFU via intramuscular route. In contrast, SUDV, BDBV, TAFV, and RESTV challenges do not cause uniform lethality in cynomolgus macaques, and the median time to death in macaques infected these viruses appears to be longer than EBOV infection; SUDV causes 50–100% lethality in 9–10 d after infection with a dose of 10^3^ PFU [6,137,139], BDBV causes 50–75% lethality in 10–13 d after infection with a dose of 10^3 −^10^4^ TCID_50_ or 10^3^ PFU [141–143], TAFV causes 60% lethality in 10–14 d after infection with a dose of >10^3^ PFU [139,144], and RESTV causes 80–100% lethality in 8–21 d after infection with a dose of 10^3^ PFU [6,145]. Considering the fact that a lethal infection in macaques can be achieved by EBOV infection with challenge doses lower than 10^3^ PFU – as low as 0.01 PFU or as little as one infectious unit – with various inoculation routes [146–149], SUDV, BDBV, TAFV, and RESTV seem to be inherently less virulent than EBOV in macaques. Parenthetically, RESTV seems to be a quite unique virus among ebolaviruses and is presumably an animal pathogen given its distinct virulence in macaques and humans. In addition to NHP studies, a longer duration of disease after experimental infection of SUDV and BDBV compared to EBOV has also been reported in a ferret model [78,150,151].

**Table 1.** Comparison of fatality rates and time to deaths in animal models infected with different ebolaviruses

	EBOV				SUDV	BDBV	TAFV	RESTV	Refs.
	Mayinga	Kikwit	Makona (early)	Makona (late)					
NHPs	100% (5–7 d)	100% (3–5 d)	100% (4–10 d)	100% (8–16 d)	50–100% (9–10 d)	50–75% (10–13 d)	60% (10–14 d)	80–100% (8–21 d)	[6,136–139,141–145,192,194,198]
Ferrets	na	100% (6 d)	100% (6–7 d)	na	100% (6–9 d)	100% (8–9 d)	na	na	[78,150,151,198,199]
HIS mice*	87–100% (8–20 d)	na	50–56% (13–22 d)	100% (9–11 d)	71% (11–19 d)	29% (17–18 d)	18% (11–13 d)	20% (13–20 d)	[93,94,96–98]
IFNAR^−/-^ mice	100% (5–6 d)	0–7% (6 d)	0–40% (9 d)	0%	5–100% (6–11 d)	0%	0%	0%	[90,91,192,193,199]

Virus inoculation route and dose varied among each publication. na: not available.

*Except for NSG-huPBL mice.

**Table 2.** Comparison of pathogenic functions of viral proteins among ebolaviruses

Protein	Pathogenic function	EBOV	SUDV	BDBV	TAFV	RESTV	Refs.
RNP complex	Genome replication/transcription*	+++	na	na	na	+	[158,160]
GP	NF-κB activation	+++	na	na	na	+	[127–131]
VP35	Inhibition of type I IFN induction	+++	+++	+++	+++	+++	[101–110,166–169]
VP24	Inhibition of ISG expression	+++	na	+	na	+++	[114–117,170]

na: not available.

*Assessed by ebolavirus minigenome system.

Notably, recently developed humanized mouse model, HLA-A2-transgenic NOD-scid-IL2γ receptor-knockout mice reconstituted with human hematopoiesis (huNSG-A2), showed a distinct susceptibility to ebolaviruses belonging to each distinct species, and the infection recapitulated species-specific CFRs in humans; EBOV, SUDV, BDBV, TAFV, and RESTV infections caused lethality of 92.3%, 71.4%, 28.6%, 18.2%, and 20% in mice, respectively [98]. This suggests that HIS greatly contributes to ebolavirus species-specific pathogenesis.

### Replication ability

Similarly to EVD, a correlation of high viremia titer and fatal disease outcome is commonly observed in SUDV disease (SVD) patients [52] and BDBV disease (BVD) patients [152]. However, very limited but some pathological examinations of postmortem specimens of fatal EVD, SVD, and BVD patients have shown that replication of SUDV and BDBV in human cells/organs might be less efficient than EBOV [54,153,154]. A previous comparative histopathological analysis showed that, whereas extensive distribution of EBOV antigen and virus inclusions composed of aggregates of viral nucleocapsids was detected in liver from EVD fatalities, no virus inclusions and the least amount of virus antigen distribution were found in liver from BVD fatalities, and SUDV infection was somewhat intermediate (Figure 3) [54]. Notably, the level of ALT, a biochemical marker for hepatocyte integrity, in fatal SVD cases was below 100 U/L [155], which is significantly lower than that generally seen in fatal EVD cases with around 500 U/L (often more than 1000 U/L) [42,156]. Moreover, while disease severity is often correlated with blood ALT levels in the EVD patients, comparable levels of ALT were observed between fatal and nonfatal cases of SVD [155]. These clinical insights suggest that abilities for replication and/or causing tissue damage in liver might differ between EBOV and other ebolaviruses.

Consistent with lower levels of replication found in postmortem specimens of fatal BVD, a reduced replication ability of BDBV was also found in an *in vitro* study using human peripheral blood mononuclear cells (PBMCs). The study showed that, whereas EBOV titer in supernatants of infected PBMCs peaked in 5 d, BDBV titer peaked at 8 d postinfection with a titer approximately 1-log lower than that of EBOV [157]. Interestingly, electron microscopic analysis showed the accumulated nucleocapsids in macrophages infected with BDBV, suggesting that BDBV can infect and replicate in human macrophages but cannot be released as efficiently as EBOV (Figure 3).

A slower and lower growth ability *in vitro* was also found in apathogenic RESTV compared to EBOV using Vero or Vero E6 cells [158,159]. Moreover, several studies using an ebolavirus minigenome system, which allow to model ebolavirus replication/transcription without using infectious virus in biosafety level 4, have suggested that the polymerase complex of RESTV exhibited a lower ability of viral RNA synthesis than that of EBOV (Table 2) [158,160]. Interestingly, supplying of EBOV RNP complex proteins, such as NP, VP35, VP30, and L, in the RESTV minigenome assay system produced a higher reporter transcription activity than using RESTV RNP complex [160]. Although it has been considered that ebolaviruses share a common strategy for their genome transcription/replication, thus the same viral proteins are required for these processes, the performance of the RNP complex for driving efficient genome transcription/replication might vary among ebolaviruses.

The intriguing difference between pathogenic and apathogenic ebolaviruses can be found in their distinct replication characteristics in parenchymal cells of the liver, which the primary target organ of EBOV replication *in vivo*. The immunohistochemistry analysis using immunodeficient mouse models demonstrated the wide distribution of EBOV antigen in the liver, by infection of hepatocytes, endothelial cells, and Kupffer cells, which are the first target cells of the liver. This is in stark contrast to RESTV replication, which has been noted to be very limited in the liver and mostly restricted to Kupffer cells (Figure 3) [96,98,159]. Consistent with this, a few *in vitro* studies have shown that RESTV replicates in primary human macrophages [130] and human monocytic leukemia THP-1 cells [161] similarly to EBOV, but not as efficient as EBOV in human hepatocellular carcinoma Huh7 cells at early phase of infection [162]. Interestingly, this distinct replication pattern in liver between pathogenic EBOV and apathogenic RESTV is also observed in a comparative study between lethal MA-EBOV and non-lethal WT-EBOV infections in Syrian golden hamsters [88]. These findings strongly suggest that the ability of virus to spread from macrophages to parenchymal cells is a key signature of pathogenic ebolaviruses.

The efficient viral replication in liver is not only responsible for its systemic spread but also the cause of significant hepatocyte necrosis manifested as severe liver damage in EVD fatalities and EBOV-infected animals. Importantly, dysfunction of the liver contributes to coagulation disorders – one of the key pathological features in EVD – due to impairment in the synthesis of important clotting factors. The correlation of viral replication in the liver and disease severity in Ebola disease pathogenesis has been widely acknowledged; however, experimental evidence to precisely compare ebolavirus replication ability in human hepatic cells is not currently available. Thus, it remains unclear whether the less efficient replication of less virulent/apathogenic ebolaviruses in liver is due to their respectively poorer inherent abilities to infect or replicate in hepatocytes or due to an unknown host antiviral mechanism(s) that interferes with viral spread from macrophages to hepatocytes. Basic characterization of viral replication ability in hepatocytes (e.g. human primary hepatic cells) is necessary to understand species-specific pathogenesis of ebolaviruses.

### Induction of uncontrolled pro-inflammatory responses

The activation of pro-inflammatory responses is considered as a common feature of fatal Ebola diseases;however, the types of cytokines/chemokines upregulated by the infection seem to be varied among viruses belonging to each distinct species. For example, the expression levels of TNF-α and IFN-γ, which have been found to be increased in EVD fatalities, were shown to be low in the SUDV infection in humans [163]. Moreover, a previous clinical study that compared cytokine expression profiles between samples from acute phase (collected within 11 d from the onset of illness) received from both BVD fatalities and survivors, and convalescent phase from BVD survivors, showed significantly lower IL-1α, IL-1β, IL-6, and TNF-α levels in acute samples than in convalescent samples (Figure 3) [152]. The levels of these cytokines expressed in BVD patients were even lower than those in the healthy control group, suggesting that unlike EBOV, BDBV infection might down-regulate these cytokine expressions in humans. A distinctive pattern of cytokines/chemokines expression induced by BDBV infection was also found in *in vitro* study [157]. BDBV infection in human PBMCs induced the expression of IL-1β, MIP-1α, TNF-α, and MCP-1 at significantly lower levels than EBOV infection. In particular, very low level of IL-1β (30–70 pg/mL) was produced in BDBV-infected PBMCs, in contrast to the EBOV infection that induced IL-1β with a peak concentration >2500 pg/mL. Intriguingly, fever with above 38°C was less common in the 2012 BVD outbreak; only 38.9% of BVD patients showed high fever during hospital stay [29]. This atypical clinical manifestation of BVD might be related to the distinct characteristic of BDBV for the regulation of pro-inflammatory responses.

A previous *in vitro* study using virus-like particles containing RESTV GP demonstrated that, unlike EBOV GP, RESTV GP does not trigger TLR4 signaling (Table 2) [130]. In agreement with this, a significantly weaker cellular response was observed in human primary monocytes or monocyte-derived macrophages (MDMs) infected with RESTV, in contrast to the strong activation of MDMs by EBOV infection shown as a markedly elevated level of pro-inflammatory genes [130,164]. These results suggest that macrophage activation mediated by GP via interaction with TLR4 might be a critical factor that determines the ebolavirus pathogenicity. On the other hand, while TLR4 inhibitors showed efficacy in the EBOV mouse model, TLR4 knockout mice still exhibited full susceptibility to the lethal MA-EBOV infection [165]. This suggests that there might be an additional factor(s), which critically involves in detrimental pro-inflammatory responses in EVD. Further analysis will be needed in order to not only precisely characterize pro-inflammatory activation profiles for each ebolavirus infection, but also define the molecular mechanisms underlying uncontrolled pro-inflammatory responses seen in Ebola diseases.

### Disturbance of functional immune responses

IFN antagonisms mediated by EBOV VP35 and VP24 have been well-characterized by a number of *in vitro* studies as described in Section “Molecular pathogenic mechanisms of EBOV infection”. Thus, the function of these viral proteins from other ebolaviruses has been closely studied in order to find a clue that determines differing pathogenicity among ebolavirus species. However, the apparent difference in terms of the type I IFN-antagonism between EBOV and other ebolaviruses has not been well-defined. It was shown that VP35 proteins from SUDV, BDBV, TAFV [166,167], and RESTV [102,166–169] are all able to inhibit type I IFN induction in the same manner as EBOV VP35 (Table 2). Moreover, whereas WT-EBOV infection becomes lethal in IFNAR^−/-^ mice or in mice treated with antibodies against IFN-α/β, the BDBV, TAFV, and RESTV infections still fail to cause lethal illness in IFNAR^−/-^ mice [90,91]. These observations suggest that, although the inhibition of the host type I IFN production – presumably solely mediated by VP35 – is critical for ebolaviruses to successfully and efficiently initiate replication in the host, this function is not the main virulence determinant among ebolavirus species.

On the other hand, species-specific difference might exist in their abilities for inhibition of type I IFN-signaling pathway as well as ISGs expression. Schwards et al. demonstrated a lower ability of BDBV VP24 to bind KPNA (KPNA1, KPNA5, and KPNA6) compared to EBOV VP24, consistent with a decreased inhibitory effect of BDBV VP24 on the expression of ISGs [170] that might explain the lower virulence of BDBV than EBOV. Moreover, a previous *in vitro* study using infectious EBOV and RESTV demonstrated that, whereas the expression of a number of ISGs was down-regulated in IFN-treated Huh7 cells with EBOV infection, RESTV infection failed to down-regulate those ISG expression [162]. This suggests that RESTV has a diminished capacity to counteract host IFN-signaling; however, this mechanism cannot be explained by its VP24’s function; although some amino acid differences were found between RESTV VP24 and EBOV VP24 [171–173], RESTV VP24 has an intact KPNA-binding capability or ISG inhibition (Table 2) [170]. Furthermore, it was shown that STAT1^−/-^ mice infected with RESTV survived with disease signs appearing slower and milder than those infected with EBOV [92,174], suggesting that differing pathogenicity between EBOV and RESTV cannot be solely explained by mechanism(s) underlying inhibition of the Jak/STAT-signaling pathway. Aside from the IFN-antagonistic functions of VP35 and VP24, their abilities to block DC maturation will be interesting to compare among ebolaviruses, which may shed the light on the species-specific immune disturbance.

A possible species-specific difference might exist in host adaptive responses against ebolavirus infections; while elevated IgG levels in non-survivors were rarely seen during the disease course of EVD [55,61,70], high IgG titers were observed in most of the BVD patients including nonsurvivors, corresponding to high virus antigen titers [152]. This finding suggests that BDBV infection may induce stronger humoral immunity than EBOV. In addition, Nehls et al. recently reported that secretory vesicles containing EBOV GP, so-called EBOV GP-virosomes, have the ability to capture neutralizing antibodies and also showed that the efficiency of the virosome formation of GPs from other ebolaviruses was lower than EBOV GP [175]; however, the practical contribution of this mechanism to viral pathogenesis has remained elusive. More experimental evidence will be needed to understand the species-specific difference in host adaptive immunity.

## Pathogenesis of EBOV-Makona variant

As discussed above, ebolavirus species-specific distinctions in pathogenicity in humans is one of the intriguing unsolved research topics in the filovirus field. Besides, the virulence of novel EBOV variant Makona, which was responsible for the 2013–2016 West Africa EVD outbreak, the largest EVD outbreak ever documented, is an emerging compelling question to be addressed for a better understanding of the EVD outbreak and EBOV pathogenesis. In this section, we discuss the biological and pathogenic differences between the EBOV-Makona variant and previously identified EBOV variants in humans based on current findings.

### Did EBOV-Makona variant acquire enhanced virulence during the outbreak?

EBOV-Makona variant was responsible for the 2013–2016 EVD outbreak that widely spread from Guinea to other countries in Western Africa, ending up a total of >28,600 human infections and >11,300 deaths [5]. During the outbreak, the number of human infections increased day-by-day with unprecedented scale, which had given rise to fears that the virus adapted to humans due to extensive human-to-human transmission events, resulting in the acquisition of enhanced transmissibility and/or virulence in humans. The discussion on the basis of sequencing analyses using available EBOV-Makona isolates was therefore primarily focused on (1) whether the EBOV-Makona is mutating more rapidly than usual and (2) whether there is any mutation(s) in the EBOV-Makona genome that possibly plays a significant role in host specificity, viral fitness, transmissibility, or virulence. Various genome analyses of more than 1500 full-length EBOV-Makona sequences – approximately 5% of those infected – have confirmed the accumulation of numerous mutations in later isolates compared to the first isolate from Guinea [176]. However, the mutation rate, which is expressed as the number of mutations per site per year, of the EBOV-Makona during this outbreak was shown to be similar to the previous outbreaks [177–181].

The large-scale sequence data clearly showed the presence of two major lineages; the first one includes isolates sampled from Guinea during the early phase of the epidemic, and the second one contains a majority of isolates sampled from all of the affected countries during the later phase of the epidemic [176,182,183]. Among all nonsynonymous mutations discovered in the viral genome of EBOV-Makona isolates in the second lineage, an amino acid substitution (Ala to Val) on GP at residue 82 (GP-A82V) was most intensively studied for its possible involvement in viral pathogenic characteristics. This was due to the emergence of the A82V mutation at the branch point that distinguishes two lineages and was fixed in the population afterward [182–186], and residue 82 on GP is located at the receptor-binding interface [187]. Several *in vitro* studies using EBOV GP-pseudotyped virus vectors or EBOV VLP systems have demonstrated that GP-A82V promotes viral entry into human cells [182,183,188], by which the mechanisms were further identified as cathepsin B and Niemann–Pick C1 dependent [189,190]. Interestingly, the enhancement of viral entry mediated by GP-A82V was only observed in primate cells, but not in rodent, carnivore [182], or bat-origin cells [183,191], suggesting that the A82V mutation might have contributed to elevated EBOV-Makona fitness in human population.

Although the above findings also raised a hypothesis in which the virulence of EBOV-Makona in humans was enhanced due to the acquisition of GP-A82V, this hypothesis has been challenged by several *in vivo* studies. Marzi et al. infected rhesus macaques with EBOV-Makona early isolates containing GP-82A and EBOV-Makona later isolates containing GP-82V and found no statistically significant difference in survival rate, tissue/blood viral load, IFN response, serum biocheminal parameters, or coagulation parameters among the infected groups [192]. Interestingly, the animals infected with EBOV-Makona later isolates showed even relatively prolonged disease progression and reduced tissue/blood viral load compared to those infected with EBOV-Makona early isolates. Moreover, *in vitro* work indicated that later isolates of EBOV-Makona replicated in human hepatocyte Huh-7 cells less efficiently than earlier isolates [192], which was contrary to the idea that the GP-A82V mutation enhances the EBOV-Makona infectivity in human cells. These results obtained by using infectious virus strongly suggest that, although it is still possible that the GP-A82V mutation has changed the biological characteristics of EBOV-Makona to some extent, those effects were most likely canceled by other mutations in GP or other viral genes in EBOV-Makona later isolates.

A mutation that is possibly involved in the attenuated phenotype of the EBOV-Makona later isolates is an amino acid substitution (Asp to Gly) on L viral RNA polymerase at residue 759 (L-D759G) [191]. Indeed, while 40% of IFNAR^−/-^ mice died after the infection of EBOV-Makona early isolate containing 759D in L, none of the mice died by the infection of a recombinant EBOV-Makona mutant in which the amino acid position 759 on L is substituted from asparagine to glycine. Moreover, a significantly delayed time to death was observed in ferrets infected with L-D759G mutant compared to WT virus. Virulence evolution, in association with the acquisition of mutation(s), is one of the compelling topics in the field of infectious diseases that often attracts a lot of attention. However, this needs to be carefully and accurately interpreted based on the combination of genomics/phylogenomics studies and confirmatory laboratory-based studies using infectious viruses.

### Variant-specific virulence between Makona variant and previously identified variants

It is important to note that several *in vivo* studies using animal models have strongly suggested that the EBOV-Makona variant, regardless of its isolates, is inherently less virulent than historical EBOV variants that were responsible for previous outbreaks [138,193–196]. Remarkably, whereas the infection of EBOV-Mayinga, the prototype EBOV variant isolated from the 1976 EVD outbreak, resulted in 100% lethality in IFNAR^−/-^ mice as has been known, none of the infected mice succumbed to the infection of either EBOV-Makona early isolate or later isolate [192]. In addition, a delayed disease progression was observed in EBOV-Makona-infected rhesus macaques and cynomolgus macaques compared to the infection of EBOV-Mayinga variant [138,192] or EBOV-Kikwit variant, isolated from the 1995 EVD outbreak [194,195]. Of note, Jankeel et al. have recently reported that viral replication, histopathological changes (e.g. liver necrosis), and inflammation in target organs were less severe in EBOV-Makona-infected macaques compared to EBOV-Kikwit-infected macaques [196]. In the case of human infections, while the EBOV-Makona caused the largest EVD outbreak in history, the overall CFR during this epidemic was 40%, which was significantly lower than CFRs of previous EVDs caused by EBOV-Mayinga or -Kikwit (CFRs = 88% or 81%, respectively). It is difficult to definitively conclude the virulence of ebolaviruses in humans according to the reported CFRs, which are easily affected by multifactorial causes including not only pathogen-related factors but also social/environmental factors such as medical infrastructures at the outbreak sites/countries or host genetic factors affecting the immune responses against virus infection. However, these virological observations strongly suggest that the EBOV-Makona variant is very unique among previously identified EBOV variants (Figure 3). While there have been some EBOV variants isolated thus far, variant-specific virulence of EBOV has not been closely investigated. Further studies will be needed to more clearly define biological and pathogenic differences among EBOV variants.

## Conclusions and future directions

This review outlined some critical abilities of EBOV for causing severe disease in humans, including robust replication and induction of dysfunctional immune responses (Figure 3). However, virulence of other ebolaviruses should not also be underestimated. For example, although human infection with TAFV has caused only one nonfatal case, the patient showed severe Ebola disease signs (e.g. diarrhea, vomiting), central nervous system disorder, coagulopathy signs including macular rash and thrombocytopenia, and drastic weight loss – 10% of the initial weight – in 15 d [32]. Moreover, the evidence showing 20% of huNSG-A2 mice succumbing to RESTV infection [98] might suggest that RESTV may have the potential to cause disease in humans under specific conditions (e.g. immune suppression).

While this review highlighted intriguing experimental findings in differing characteristics of ebolaviruses, there are still some significant gaps in our knowledge about ebolavirus species-specific distinctions in pathogenicity at the molecular level. This is in part because, compared to EBOV, our understanding of the basic biology of SUDV, BDBV, TAFV, and RESTV has largely lagged behind. For example, although viral replication in the liver has been known to be critically involved in the pathogenesis of Ebola disease, replication of SUDV, BDBV, TAFV, and RESTV in hepatocytes (e.g. human primary hepatic cells) has not been fully characterized yet. Head-to-head comparison of replication abilities of EBOV with other ebolaviruses in various cell types will provide fundamental information on ebolavirus biology, which is also necessary to evaluate the pathogenic characteristics of each ebolavirus. The application of virus life cycle modeling systems, such as minigenome and transcription/replication-competent VLP systems, would also provide valuable insights into the molecular mechanisms of differing replication characteristics among ebolaviruses.

*In vivo* analysis using recombinant chimeric ebolaviruses between pathogenic EBOV and apathogenic/less virulent ebolaviruses generated by reverse genetics will also facilitate the identification of ebolavirus virulence determinant(s). Interestingly, a chimeric EBOV possessing RESTV GP replicated in macrophages similarly to the parental EBOV but decreased infection of hepatocytes in infected IFNAR^−/-^ mice [159], suggesting that the RESTV GP may have a deficient interaction with hepatocyte-specific ebolavirus receptor(s), which has not been characterized yet. On the other hand, a chimeric RESTV possessing EBOV GP did not restore the ability of the parental RESTV to replicate in hepatocytes, implying that the ability of virus to spread from macrophages to parenchymal cells – a key signature of pathogenic ebolaviruses (Figure 3) – seems to be defined by multifactorial determinants. The rapidly developing single-cell RNA sequencing technology would be one of the most powerful approaches that enable not only precise identification of virus-infected cell type(s) in target organs, but also a comprehensive understanding of differing host immune responses against each ebolavirus infection at the single-cell level.

One of the significant limitations in conducting comparative pathogenesis studies among ebolavirus species was a lack of animal models that recapitulate differing Ebola diseases in humans. Using recently developed HIS mouse models in future comparative studies will facilitate the understanding on the interaction between virus and host immunity in each Ebola disease at the molecular level. In addition, data sets from several large-scale analyses, including specificity determining positions analysis [173], global phosphoproteomic analysis [197], and multi-platform ’omics analysis [58], have become available. Utilizing these comprehensive data sets, in combination with authentic virological approaches including utilization of reverse genetics for the in-depth understanding of molecular virology aspects, may help to define different characteristics among ebolaviruses. Further studies examining species-specific and variant-specific virulence of ebolaviruses at the molecular level will facilitate not only a better understanding of the basic biology of genus *Ebolavirus* but also the development of therapeutics against well-focused pathogenic mechanisms of each Ebola disease.
